# Real-World Evidence Evaluation on the Lipid Profile, Therapeutic Goals, and Safety of the Fixed-Dose Combination of Rosuvastatin/Ezetimibe (Trezete®) in Dyslipidemia Patients

**DOI:** 10.1155/2022/9464733

**Published:** 2022-09-10

**Authors:** Joel Rodríguez-Saldaña, Francisco Padilla-Padilla, Ernesto G. Cardona-Muñoz, Yulia Romero-Antonio, María Marcela Arguedas-Núñez, José G. Sander-Padilla, Alberto Martínez-Muñoz, Laura A. Lugo-Sánchez, Ileana C. Rodríguez-Vazquez, Jorge González-Canudas

**Affiliations:** ^1^Resultados Médicos Desarrollo e Investigación S.C., Pachuca, Hidalgo, Mexico; ^2^Clinical and Interventional Cardiology, Ajijic, Jalisco, Mexico; ^3^Private Practice, Puerto Vallarta, Jalisco, Mexico; ^4^Laboratorios Silanes S.A. de C.V., México City, Mexico; ^5^IMSS-Centro Médico Nacional Siglo XXI, México City, Mexico

## Abstract

**Introduction:**

Cardiovascular diseases are the leading cause of death worldwide. The combination of statins and cholesterol-absorption inhibitors promotes the decrease in risk factors, such as high concentrations of LDL (low-density lipoproteins). The aim of the study was to evaluate changes in the lipid profile and the effect on therapeutic goals, as well as the safety of dyslipidemia patients treated with Rosuvastatin/Ezetimibe (Trezete®).

**Materials and Methods:**

A real-world evidence study was conducted with retrospective data collection through a review of clinical records from dyslipidemia patients treated with Trezete® in routine medical practice. Clinical records included results of biochemical markers before treatment and at least one follow up between weeks 8 and 16.

**Results:**

The study included 103 patients' clinical records (55.4% men) with a mean age of 56.0 ± 13.0 years. More than 57% of the patients had mixed dyslipidemia and a median disease progression of 3.1 (IQR, 1.5; 9.1) years. Regarding LDL concentrations, 72.8% of the patients achieved therapeutic goals according to cardiovascular risk (CVR), which was statistically significant. Similarly, 94.1% achieved goals for total cholesterol (<200 mg/dL) and 56.0% for triglycerides (<150 mg/dL), a *p* value <0.001. No cardiovascular events were observed.

**Conclusion:**

Trezete® shows an important clinical impact on CVR-related target markers during the treatment of dyslipidemia patients. It is relevant to mention that a significant percentage of patients achieved therapeutic goals during the first months of treatment. Fixed-dose combination therapy has shown to be as safe as monotherapy treatment. ClinicalTrials.gov Identifier: NCT04862962.

## 1. Introduction

In recent years, cardiovascular diseases have become the leading cause of death worldwide, with atherosclerotic-type alterations being the most predominant and considered an important risk factor for the appearance of future complications [1].

Dyslipidemias are asymptomatic diseases characterized by alterations in normal blood lipid levels, including variants such as increased low-density lipoprotein cholesterol (LDL-c) and/or triglycerides and/or decreased high-density lipoprotein cholesterol (HDL-C) [2]. These disorders are related to environmental and genetic factors which interact to determine lipid levels in a person [3]. The most prevalent dyslipidemias identified in the Mexican population, according to national surveys, are hypercholesterolemia (total cholesterol ≥200 mg/dL), hypertriglyceridemia (triglycerides ≥150 mg/dL), and hypoalphalipoproteinemia (HDL-c <40 mg/dL), the last one being the most frequent, affecting up to four out of five adults [4].

Regarding the treatment for these alterations, the economic cost is significantly high. However, it represents a much lower cost in comparison to those generated by cardiovascular complications that can be prevented [4]. Although lifestyle considerations play a key role in the treatment of dyslipidemia, the use of pharmacological therapy contributes significantly to the control of risk factors. The use of statins is one of the main options, due to their effect on the reduction of cholesterol synthesis [5, 6].

Other important medications include cholesterol-absorption inhibitors, such as ezetimibe. Whereas, triglyceride-reducing drugs consist mainly of fibrates and niacin [7].

The 2018 guidelines from the American College of Cardiology/American Heart Association (ACC/AHA) emphasized the use of statin therapies for the management of blood cholesterol [8] highlighting the findings of numerous studies on the benefits of moderate and high-intensity statin therapy, such as the reduction in the risk of presenting atherosclerotic cardiovascular disease [9].

Rosuvastatin is a new generation HMG-CoA reductase enzyme inhibitor, with low penetration of the extrahepatic tissue. It has low potential for interactions with cytochrome P450 3A4 (CYP3A4) and a substantial capacity to reduce LDL-c [10]. This therapy is currently prescribed to lower levels of total cholesterol, LDL-c, and triglyceride, and increased HDL-c levels in patients with primary hypercholesterolemia, mixed dyslipidemia, and homozygous familial hypercholesterolemia [11].

Ezetimibe is a selective inhibitor of cholesterol absorption. A standard dose of 10 mg/day reduces LDL-c by 15–20% when used as monotherapy. Its combination with statins has shown to have a greater effect, allowing patients to reach the recommended target numbers [12]. Studies suggest that adding ezetimibe or fenofibrate to statins could further reduce the levels of LDL-c, total cholesterol, and triglycerides, and potentially increase HDL-c [13].

Recent studies have shown that the combined use of ezetimibe, which inhibits cholesterol absorption from external sources, and statins, whose main action is the inhibition of cholesterol synthesis in the liver, has an important effect on the treatment of dyslipidemias, mainly in the reduction of markers such as LDL-c, due to the complementary activity exhibited by these two drugs [14].

Currently, fixed-dose combinations (FDC) have allowed an improvement in the treatment of dyslipidemia patients, since their main function is to reinforce lipid-lowering therapy, without the need to ingest a larger number of tablets, which gives the treatment a greater degree of complexity. These combinations have shown a significant increase in treatment adherence compared to monotherapies, as well as a higher success rate for LDL-c targets. Rosuvastatin/ezetimibe FDC has shown to be more effective in lowering LDL-c levels, as well as atherosclerotic plaque, and more importantly, it has been associated with a lower incidence of cardiovascular events compared to statin monotherapy. However, a few studies researched the effect of this FDC in uncontrolled environments, within routine medical practice. These studies provide information over a longer follow-up for these types of patients [15]. The aim of the present study was to conduct a real-world evidence evaluation of lipid profile changes, the effect on therapeutic goals, as well as the safety of dyslipidemia patients treated with rosuvastatin/ezetimibe (Trezete®) in routine medical practice.

## 2. Materials and Methods

### 2.1. Study Design and Study Population

A real-world evidence, observational, multicenter study was conducted in three centers in different states of Mexico, with retrospective data collection, through the review of clinical records of dyslipidemia patients treated with the FDC rosuvastatin/ezetimibe (Trezete®, 10 mg/10 mg or 20 mg/10 mg). Clinical records of patients who met the inclusion criteria and had results for biochemical markers before treatment and at least one follow up between weeks 8 and 16 were selected during 2020 and 2021.

### 2.2. Eligibility Criteria

**Figure 1:**
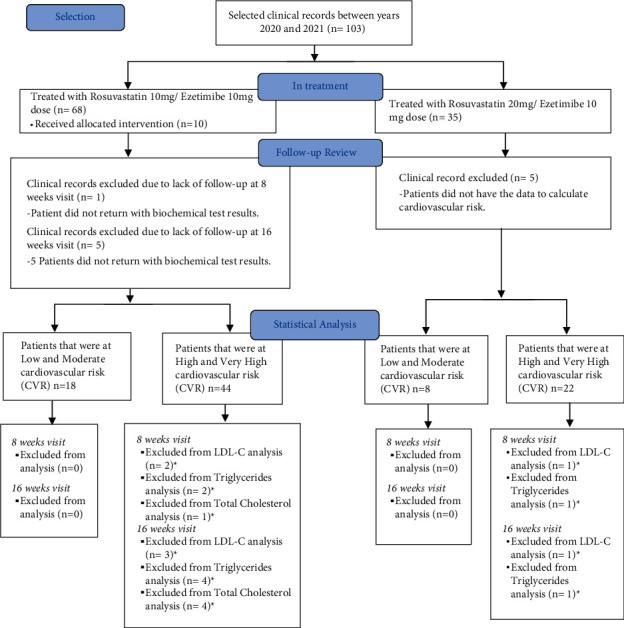
Flowchart of selection and analysis of patient clinical records. ^*∗*^ Patients did not have data for this lipid marker.

Clinical records were included if patients were 18 years or older, diagnosed with dyslipidemia, provided documented treatment with rosuvastatin/ezetimibe FDC, and a safety questionnaire on at least two occasions. Clinical records were excluded when concomitant consumption of some other statin or fibrate was documented during rosuvastatin/ezetimibe treatment. Medical history information was recorded, including a general clinical review, family history, cardiovascular risk factors, and a physical examination. Also, anthropometric measurements (weight, height, waist circumference, and body mass index), vital signs (blood pressure, heart rate, respiratory rate, and temperature), and clinical laboratories requested by the treating physician were obtained. The flow chart for the selection of clinical records and data analysis is shown in Figure 1.

### 2.3. Laboratory Analysis

The results and frequency of biochemical tests required by the treating physician for each patient were reviewed, generating a record of these for subsequent comparative analysis. All biochemical tests documented in a patient's clinical record with dates consistent with their treatment initiation and follow-up visits were considered. All the patients attended certified external clinical laboratories for blood sample collection and analysis.

### 2.4. Lipid Profile and Therapeutic Goals

Patients who had results of baseline lipid markers and at least one follow up were included. The lipid profile consisted of total cholesterol (TC), triglycerides (TG), low-density lipoprotein (LDL-c), very low-density lipoprotein (VLDL), and high-density lipoprotein cholesterol (HDL-c). The Atherogenic Index (AI) was calculated with the formula AI = Log(TG/HDL-c) and non-HDL cholesterol was calculated with the formula non-HDL = TC—HDL-c.

Therapeutic goals for LDL-c and non-HDL-c levels were established according to the cardiovascular risk for each patient and by the Mexican Clinical Practice Guide for the diagnosis and treatment of dyslipidemias [16]. The goals for total cholesterol and triglycerides were established based on the 2019 European Society of Cardiology/European Atherosclerosis Society (ESC/EAS) guidelines on the treatment of dyslipidemias [17]. HDL-c concentrations were not considered within the therapeutic goals, because the follow-up time was not long enough to observe significant changes, and there was no information on lifestyle interventions.

### 2.5. Cardiovascular Risk Calculation

Patients were classified according to their cardiovascular risk, which was obtained using the PAHO/WHO—PAHO calculator [18]. This calculator uses information such as the patient's history of cardiovascular diseases, chronic degenerative diseases (diabetes and hypertension), as well as systolic blood pressure, age, sex, smoking background, and cholesterol levels.

### 2.6. Safety

The records of adverse events reported within a patient's file were reviewed in order to assess safety. The Naranjo algorithm was used to explore the cause-effect relationship (causality and imputability) of adverse events documented in clinical records by the treating physician.

### 2.7. Ethical Conduct

All patients signed informed consent for the use of their information. The study protocol was approved by an ethics committee (NO. CEI-000002), a research committee, and the Ministry of Health in Mexico (COFEPRIS).

### 2.8. Statistical Analysis

The review of the clinical records was performed at convenience. However, it was considered that at least 87 patient clinical records were necessary to meet the safety objective, taking into account 3% for one of the rarest adverse events (myalgia), a statistical power of 90%, and an alpha error probability of 0.03.

Data are expressed as mean ± standard deviation (SD) for normal distribution quantitative variables, median, and interquartile range (IQR) for variables with nonnormal distribution, and as percentages for categorical variables. Differences between time points were assessed using the Wilcoxon rank sum test, McNemar's test, and paired Student's *t*-test. Differences between doses were evaluated using Student's *t*-test, chi-square test, and Mann–Whitney *U* test. Statistical significance was accepted with a *p* value <0.05. Analysis was performed with IBM SPSS Statistics for Windows, version 28 (IBM Corp., Armonk, N.Y, USA).

## 3. Results

Clinical records of 103 patients (55.4%, men) with a mean age of 56.0 ± 13.0 years were included. The median body mass index (BMI) was 28.46 kg/m^2^ (IQR, 26.53; 31.84), indicating that most patients were overweight. At the beginning of treatment, 57.3% of the patients had mixed dyslipidemia and a median disease progression of 3.1 (IQR, 1.5; 9.1) years. The most prevalent comorbidities were diabetes (55.3%), arterial hypertension (56.3%), and heart disease (11.7%). About 32.1% of patients had obesity. Concomitant medications were observed in 64 (62.1%) subjects, insulin and angiotensin receptor antagonists being the most used (26.2%), followed by biguanides and beta-blockers (18.4 and 16.5%, respectively). The complete baseline status is shown in Table 1.

### 3.1. Lipid Profile and Therapeutic Goals

Significant changes were observed starting from visit 1 (2.03 ± 0.55 months of follow up) and maintained until visit 2 (4.22 ± 0.83 months of follow up). Changes in lipid levels were observed in practically all lipid profiles, which was statistically significant (*p* ≤ 0.001), except for HDL-c, which showed a minimal increase during follow up (Table 2).

When evaluating the proportion and changes in concentrations between follow-up visits versus baseline (Table 3), subjects achieved significant reductions in LDL-c and non-HDL-c levels. A percentage of success between 50–55% was observed for both markers, with a statistically significant difference (*p* ≤ 0.001) from the follow-up at 8 weeks, amongst patients whose reduction was >50% or <50% for LDL-c (88.6 (IQR, −103.1; −67.2) and −20.9 (IQR, −40.4; 0.0), respectively) and for non-HDL-c (−60.1 (−67.0; −57.4) and -29.8 (−37.2; −5.8), respectively).

**Figure 2:**
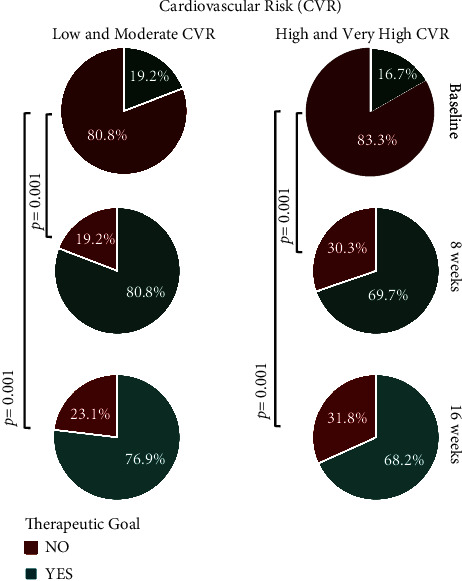
Proportion of patients who met therapeutic goals according to the degree of cardiovascular risk (CVR) at 8 and 16 weeks, compared to baseline. A *p* value of <0.05 was taken as statistically significant.

An analysis of the data obtained for the LDL-c marker was carried out to identify the percentage of patients who managed to meet the goals determined according to CVR, which was established by the Mexican Clinical Practice Guide for the diagnosis and treatment of dyslipidemias. This evaluation showed that 72.8% of patients achieved the mentioned goal, this being statistically significant with *p*=0.001 (Figure 2).

At the 8-week visit, 22.8% and 50% of the patients with CVR (low–moderate and high–very high, respectively) had achieved their goal for LDL-c, while 55.4% of the 92 patients whose CVR was calculated achieved the established goal for non-HDL-C (Table 4).

**Figure 3:**
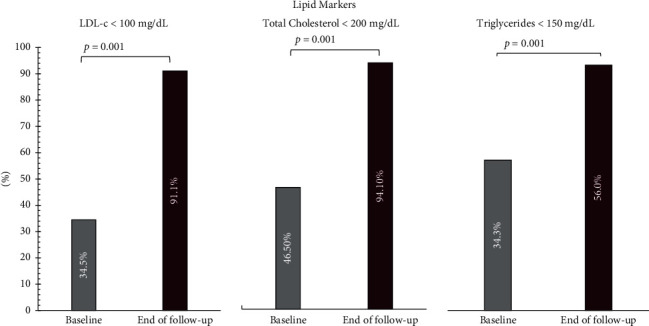
Percentage of patients that achieved therapeutic goals for total cholesterol, triglycerides, and LDL-c at the end of the follow up, compared to baseline. A *p* value of <0.05 was taken as statistically significant.

At the end of the follow up, 94.1% of the study population had achieved the therapeutic goal for total cholesterol (<200 mg/dL), 56.0% for triglycerides (<150 mg/dL), and 91.1% for LDL-c (<100 mg/dL), regardless of when the goal was achieved (8 or 16 weeks), with *p* < 0.001 (Figure 3). Results are shown in Table 5.

Additionally, an analysis was performed to identify differences in the achieved therapeutic goals according to CVR between doses (10 mg/10 mg and 20 mg/10 mg), observing no differences in the proportion of patients who reached the established goal or in the median change in lipid marker concentrations over time. However, the comparison between the proportion of patients who achieved the therapeutic goal according to CVR at 8 and 16 weeks versus baseline within the same dose was statistically significant, as well as the change in the median concentration over time in the three lipid markers, with a reduction of more than −70.0 mg/dL and −116.0 mg/dL for low and moderate CVR in the doses 10 mg/10 mg and 20 mg/10 mg, respectively.

### 3.2. Safety

A descriptive analysis of adverse events (AE) displayed during treatment was performed (Table 6). A total of 24 AEs was observed in 20 patients (50.0% men) with a mean age of 60.1 ± 13.9 years. The only probable AE was myalgia in one patient. The most prevalent AEs were elevated liver enzymes (12.5%), COVID-19 disease (12.5%), and COVID-19 pneumonia (8.3%) being mostly nonserious (91.7%), moderate severity (62.5%), improbable causality (54.2%), and unexpected (70.8%). There were no cardiovascular AEs documented during the follow up.

## 4. Discussion

Since LDL-c has a key role in the formation and progression of atherosclerosis [19], LDL-c levels are considered a major cardiovascular disease risk factor. Pharmacological therapy for at-risk patients is a sound path to reduce this biomarker [20].

Statins and cholesterol-absorption inhibitors are two of the most prescribed drugs for dyslipidemia therapy; their ability for reducing LDL-c levels has been vastly proven [17]. However, studies such as SHARP [21] and IMPROVE-IT [22] have demonstrated that the combination of statins and cholesterol-absorption inhibitors, compared with monotherapy, results in a greater reduction in LDL-c levels, without a significant increase in adverse events.

The present study showed significant efficacy and safety results with a fixed-dose combination of rosuvastatin/ezetimibe over lipid profile markers such as total cholesterol, triglycerides, and LDL-c. This demonstrates an important clinical impact on target markers for the treatment of dyslipidemia patients with different degrees of CVR. Results showed that about 80% of patients with low or moderate CVR achieved their LDL-c therapeutic goal at 8 weeks and maintained it until 16 weeks. Meanwhile, approximately 70% and 77% of patients with high and very-high CVR achieved their goals for LDL-C and non-HDL-c, respectively. Comparably, in the MRS-ROZE study [23] and the I-ROSETTE study [24], randomized, double-blind, multicenter, comparative phase III clinical trials evaluated the efficacy and safety of a fixed-dose combination of rosuvastatin/ezetimibe, compared to rosuvastatin alone in 407 (MRS-ROZE) and 396 (I-ROSETTE) patients with primary hypercholesterolemia for 8 weeks. A higher reduction of LDL-c, total cholesterol, and triglycerides in the fixed-dose combination group was observed.

It is important to highlight that more than 90% of patients achieved the final therapeutic target for total cholesterol and LDL-c established by the recommended guidelines for dyslipidemia. These results were statistically significant for both the 8- and 16-week follow up. In this way, it was possible to observe higher percentages of reduction in concentrations not only of the variable of interest (LDL-c), but also in parameters such as triglycerides, obtaining changes of over 20%. These results were higher than those reported in previous studies, where the percentage of change was around 13% for the rosuvastatin/ezetimibe combination. As reported in previous studies, where the maximum reduction was around 3%, no significant changes were observed in HDL-c (mean reduction of <1%), which may be due to the fact that this marker usually presents results with longer follow-up and through additional interventions, such as lifestyle changes, which were not assessed in our study. In relation to adverse events, symptoms of interest such as myalgia occurred in a lower proportion (4.2%) compared to what was reported by previous clinical studies. [25, 26]

Finally, the [27] study conducted by Vattimo et al., a randomized, open-label, multicenter, noninferiority phase III clinical trial (rosuvastatin/ezetimibe vs. simvastatin/ezetimibe), evaluated the efficacy and safety of the rosuvastatin/ezetimibe combination (Trezete®) in 129 patients with high cardiovascular risk and primary hypercholesterolemia or mixed dyslipidemia during 9 weeks. They concluded that the combination rosuvastatin/ezetimibe was not inferior to the use of the simvastatin /ezetimibe, since both showed a significant reduction in LDL-c, non-HDL-c, triglycerides, and apolipoprotein B cholesterol. These clinical trials reinforce the results obtained in the present study on the effect of rosuvastatin/ezetimibe FDC (Trezete®) on the lipid profile.

The main limitation of our study was that it was retrospective with information obtained from clinical records, which made it an open and uncontrolled clinical trial that lacked more robust inclusion criteria. It is important to continue the study of this FDC in a prospective way, with a longer follow-up time and a larger number of patients.

However, it is important to recognize that the study had fundamental strengths, such as a longer follow-up period for these types of patients and results that reflect the management and behavior of the rosuvastatin/ezetimibe combination in routine medical practice.

The present study is the first real-world evidence evaluation of the efficacy and safety of a rosuvastatin/ezetimibe FDC with a 16-week follow up, providing real daily management of dyslipidemia patients. Our study demonstrates that even in an uncontrolled environment, this FDC (Trezete®) presents a significant and constant achievement of therapeutic goals according to the CVR of each patient. Safety analysis showed that the AE profile of the FDC does not differ in incidence, severity, and seriousness from those presented by the monotherapy of the evaluated drugs, demonstrating the safety of the rosuvastatin/ezetimibe FDC.

## Figures and Tables

**Table 1:** Baseline demographic, clinical, and biochemical characteristics.

Variable	Total *n* = 103 (%)
Age, years (mean, SD)	56.0 ± 13.0
Gender (male %)	55 (55.4)

Anthropometric characteristics	
Weight, kg (median, IQR)	75.5 (68.4; 91.7)
Height, m (mean, SD)	1.64 ± 0.10
Waist, cm (mean, SD)	103.0 ± 14.0
Body mass index, kg/m^2^ (median, IQR)	28.46 (26.53; 31.84)

Clinical characteristics	
SBP, mmHg (median, IQR)	125.0 (112.0; 140.0)
DBP, mmHg (median, IQR)	76.0 (69.0; 81.0)
Heart rate, bpm (median, IQR)	72.0 (65.0; 82.0)
Respiratory rate, bpm (median, IQR)	18.0 (17.0; 18.0)
Temperature, ° C (median, IQR)	36.5 (35.2; 36.7)

Biochemical characteristics	
Glucose (mg/dL)	113.0 (93.0; 172.0)
Urea (mg/dL)	32.0 (27.4; 38.0)
Creatinine (mg/dL)	0.85 (0.68; 1.01)
Total cholesterol (mg/dL)	199.1 ± 60.1
HDL cholesterol (mg/dL)	38.9 (33.0; 47.0)
Non-HDL cholesterol (mg/dL)	154.2 ± 59.2
LDL cholesterol (mg/dL)	119.0 (75.4; 143.6)
VLDL cholesterol (mg/dL)	34.0 (22.0; 52.0)
Triglycerides (mg/dL)	219 (126.0; 336.0)
Atherogenic Index	0.75 (0.43; 0.94)
Sodium (mmol/L)	138.5 ± 2.8
Potassium (mmol/L)	4.4 (4.1; 4.6)
Chloride (mmol/L)	103.5 ± 2.4)
Aspartate aminotransferase (U/L)	22.0 (18.0; 26.0)
Alanine aminotransferase (U/L)	26.0 (18.0; 35.0)
Alkaline phosphatase (U/L)	86.0 (66.0; 109.7)
Gamma glutamyl transpeptidase (U/L)	34.5 (20.0; 46.0)
Total creatine kinase (U/L)	80.0 (62.0; 122.0)
Iron (g/dL)	91.1 (70.0; 114.0)
Transferrin (mg/dL)	259.0 ± 41.0
Erythrocytes (10^6/µL)	5.0 ± 0.6
Hemoglobin (g/dL)	15.1 ± 1.7
Platelets	(10^3/µl)
Leukocytes (10^3/µL)	7.1 ± 1.8
Cardiac C-reactive protein (mg/dL)	2.28 (0.83; 5.32)

Treatment dose	
Rosuvastatin 10/ezetimibe 10 mg	68.0 (66.0)
Rosuvastatin 20/ezetimibe 10 mg	35.0 (34.0)

Type of dyslipidemia	
Hypertriglyceridemia	13.0 (12.6)
Hypercholesterolemia	18.0 (17.5)
Mixed	59.0 (57.3)
Years of progression (median, IQR)	3.1 (1.5; 9.1)

Body mass index	
Normal weight	8.0 (7.8)
Overweight	51.0 (49.5)
Obesity degree I	22.0 (21.4)
Obesity degree II	7.0 (6.8)
Morbid obesity	4.0 (3.9)
Globorisk, %	9.81 ± 5.89

Comorbidities	
Diabetes	57.0 (55.3)
Arterial hypertension	58.0 (56.3)
Heart diseases	12.0 (11.7)
Alcohol	41.0 (39.8)
Smoking	9 (8.7)

Cardiovascular risk	
Low risk	14 (15.2)
Moderate risk	12 (13.0)
High risk	45 (48.9)
Very-high risk	21 (22.8)

Glomerular filtration rate	97.0 (80.0; 105.0)
Concomitant medications	64.0 (62.1)
Number of medications per patient	2.0 (0.0; 4.0)

Abbreviations: HDL: high-density lipoprotein; LDL: low-density lipoprotein; VLDL: very-low-density lipoprotein; SBP: systolic blood pressure; DBP: diastolic blood pressure; CRP: C-reactive protein; bpm: beats per minute or breaths per minute; kg: kilograms; cm: centimeters; mmHg: millimeters of mercury; mg: milligrams; dl: deciliter; U/L: international units per liter; *µ*L: microliters. Triglycerides results for two patients were omitted because they represented extreme data (1,474 and 3141 mg/dL). Data were available to calculate cardiovascular risk from 92 patients and from 99 patients to calculate the GFR (glomerular filtration rate). The PAHO/WHO—PAHO calculator was used.

**Table 2:** Changes in biochemical markers at 8 and 16 weeks of treatment.

Variable	Baseline	8 weeks	*p* ^*∗*^	16 weeks	*p* ^+^
Glucose (mg/dL)	113.0 (93.0; 172.0)	107.0 (90.0; 154.0)	0.103	112.0 (95.0; 152.0)	0.383
Urea (mg/dL)	32.0 (27.4; 38.0)	31.0 (25.0; 40.0)	0.884	31.6 (26.0; 37.0)	0.781
Creatinine (mg/dL)	0.85 (0.68; 1.01)	0.8 (0.66; 0.94)	0.029	0.80 (0.68; 0.94)	**0.030**
Uric Acid(mg/dL)	5.8 ± 1.4	5.6 ± 1.4	**0.002**	5.4 ± 1.5	**0.002**
Total cholesterol (mg/dL)	199.1 ± 60.1	132.5 ± 48.3	**0.001**	134.4 ± 47.2	**0.001**
HDL cholesterol (mg/dL)	38.9 (33.0; 47.0)	39.8 (33.2; 49.5)	0.968	38.7 (33.4; 46.29)	0.400
Non-HDL cholesterol (mg/dL)	154.2 ± 59.2	90.7 ± 48.5	**0.001**	94.2 ± 45.4	**0.001**
Triglycerides (mg/dL)	219 (126.0; 336.0)	154.0 (107.0; 218.5)	**0.001**	156.0 (108.0; 231.0)	**0.001**
LDL cholesterol (mg/dL)	119.0 (75.4; 143.6)	46.6 (38.4; 74.8)	**0.001**	51.7 (38.5; 79.0)	**0.001**
VLDL cholesterol (mg/dL)	34.0 (22.0; 52.0)	27.0 (19.5; 33.0)	**0.001**	24.0 (19.0; 33.0)	**0.001**
Atherogenic Index	0.75 (0.43; 0.94)	0.59 (0.39; 0.78)	**0.001**	0.62 (0.42; 0.75)	**0.002**

*p* ^*∗*^ baseline vs 8 weeks; *p* + baseline vs 16 weeks; *p*° 8 weeks vs 16 weeks. The nonparametric Wilcoxon rank sum test for nonnormal variables and the paired *T*-student test for data with normal distribution. ^*∗∗*^ The variable with the smallest number of subjects was VLDL-C with 81 patients for the 16th week.

**Table 3:** Proportion of subjects with >50% reduction in non-HDL-c and LDL-c.

Variable	8 weeks		*p* ^*∗*^	16 weeks		*p* ^*∗*^
	*n* = 86 (%)	∆		*n* = 83 (%)	∆	
Non-high-density lipoprotein cholesterol						
>50%	37 (43.0)	−60.1 (−67.0; −57.4)	**0.001**	38 (45.8)	−62.6 (−66.8; −56.9)	**0.001**
<50%	49 (57.0)	−29.8 (−37.2; −5.8)		45 (54.2)	−24.5 (−38.4; 2.6)	

Low-density cholesterol						
>50%	46 (54.8)	−88.6 (−103.1; −67.2)	**0.001**	43 (53.1)	−89.3 (−11.8; −62.2)	**0.001**
<50%	38 (45.2)	−20.9 (−40.4; 0.0)		38 (46.9)	−7.0 (−36.3; 3.0)	

*p* ^*∗*^; Comparison of deltas (Δ) between reduction groups (<50% vs >50%) with the Mann–Whitney *U* test for independent samples, *p*^+^; comparison of proportions at 8 vs. 16 weeks with the McNemar's test. For the LDL-c variable, information was obtained from 84 patients for 8 weeks follow up and 81 patients for 16 weeks follow up.

**Table 4:** Therapeutic goals according to cardiovascular risk.

Variable	Baseline *n* = 92 (%)	8 weeks *n* = 92 (%)	16 weeks *n* = 92 (%)	*p* ^*∗*^	*p* ^+^
Low and moderate CVR (*n* = 26)					
LDL-c <115 or <100 mg/dL	5 (5.4)	21 (22.8)	20 (22.7)	**0.001**	**0.001**

High and very-high CVR (*n* = 66)					
LDL-c <70 or <55 mg/dL	11 (12.0)	46 (50.0)	45 (48.9)	**0.001**	**0.001**
Non-HDL-c <130 or <100 mg/dL	18 (19.5)	51 (55.4)	52 (56.5)	**0.001**	**0.001**

CVR: cardiovascular risk. For the baseline visit, data were only available for 79 patients out of the 92 for whom CVR was calculated, 89 for 8 weeks and 88 for 16 weeks. *p*^*∗*^: comparison baseline vs. 8 weeks; *p*^+^: comparison baseline vs 16 weeks; the McNemar's test was used for this evaluation. The PAHO/WHO—PAHO calculator was used to calculate CVR.

**Table 5:** Proportion and deltas of patients who achieved therapeutic goals for total cholesterol, triglycerides, and LDL-c per dose at the end of follow up compared to baseline.

Variable	Baseline *n* = 102 (%)				End of follow up *n* = 102 (%)				*p*∧
	Total	10/10 *n* = 66	20/10 *n* = 35	End of follow-up total	10/10		20/10		
					*n* = 67	∆	*n* = 35	∆	
Total cholesterol (<200 mg/dl)	47 (46.5)	30 (45.5)	17 (48.6)	96 (94.1)	65 (95.6)	−79.0 (−96.0; −39.0)	31 (88.6)	−58.0 (−131.0; −8.0)	**0.001**
Triglycerides (<150 mg/dl)	34 (34.3)	18 (27.7)	16 (47.1)	56 (56.0)	33 (48.5)	−56.6 (−146.0; 11.0)	23 (65.7)	−16.3 (−121.0; 9.0)	**0.001**
LDL-c (<100 mg/dl)	30 (34.5)	17 (29.3)	13 (44.8)	92 (91.1)	61 (89.7)	−48.8 (−88.1; −15.0)	31 (88.6)	−39.0 (−90.5; −15.8)	**0.001**

For the total cholesterol variable, 102 subjects were included at the end of follow up; triglycerides had data from 100 subjects for the end of follow-up; for LDL-C, data from 102 subjects were available at the end of follow-up. *p*∧: baseline comparison Vs final follow up with the McNemar's test. The valid percentage is used for the final sample size at each visit.

**Table 6:** Description of patients and adverse events displayed during the study.

Variable	Total *n* = 103 (%)
No. of subjects who presented AE	20 (19.4)
Age, years (mean, SD)	60.1 ± 13.9
Gender (men, %)	10 (50.0)
Adverse event	24 (%)
COVID-19 pneumonia	2 (8.3)
COVID-19	3 (12.5)
Viral bronchitis	1 (4.2)
Increased liver enzyme	3 (12.5)
Atrial fibrillation	1 (4.2)
Diabetic nephropathy	1 (4.2)
Constipation	1 (4.2)
Herpes zoster	1 (4.2)
Depression	2 (8.3)
Increased amylase	1 (4.2)
Irritable bowel syndrome	1 (4.2)
Cervicitis	1 (4.2)
Dizziness	1 (4.2)
Hyperkalemia	1 (4.2)
Pharyngitis	1 (4.2)
Orthostatic hypotension	1 (4.2)
Myalgia	1 (4.2)
Urinary tract infection by *E. coli*	1 (4.2)
Seriousness	
Serious	2 (8.3)
Nonserious	22 (91.7)
Severity	
Mild	9 (37.5)
Moderate	15 (62.5)
Causality	
Improbable	13 (54.2)
Possible	10 (41.7)
Probable	1 (4.2)
Expectability	
Expected	7 (29.2)
Unexpected	17 (70.8)

Reported with frequencies (percentages). Although a hyperkalemia adverse event was reported, the median potassium concentrations were not above the normal range at any time during the follow up of patients.

## Data Availability

Data will be available from the corresponding author upon reasonable request via e-mail (jogonzalez@silanes.com.mx).

## Additional Points

The Ministry of Health in Mexico Federal Commission for the Protection against Sanitary Risks (COFEPRIS) under approval number: 213300410D0002/2021.
